# Supplementary material for: From sequence to enzyme mechanism using multi-label machine learning
Source: BMC Bioinformatics. 2014 May 19;15:150. doi: 10.1186/1471-2105-15-150 (PMC4229970; doi:10.1186/1471-2105-15-150)
Supplement: Additional file 2 — Java code of ml2db. Additional file ml2db_code.tar.gz contains the Java source code to run the multi-label machine learning experiments and save the results to database. The code’s Javadoc is included. [file 1471-2105-15-150-S2.zip › additional file 2/ml2db/utils/doc/uk/ac/ed/inf/utils/guiutils/CommandLineMenu.html]

CommandLineMenu


---


|  |  |  |
| --- | --- | --- |
| **Overview** \| **Package** \| **Class** \| **Use** \| **Tree** \| **Deprecated** \| **Index** \| **Help** | |  |
| PREV CLASS   **NEXT CLASS** | **FRAMES**    **NO FRAMES**     **All Classes** |  |
| SUMMARY: NESTED \| FIELD \| CONSTR \| METHOD | DETAIL: FIELD \| CONSTR \| METHOD |  |


---


## uk.ac.ed.inf.utils.guiutils Class CommandLineMenu

```
java.lang.Object
  uk.ac.ed.inf.utils.guiutils.CommandLineMenu
```

---

``` public class CommandLineMenu extends java.lang.Object ```

Stores the options for a command line program.

**Version:**
:   14 Dec 2007

**Author:**
:   Luna De Ferrari luna.deferrari-at-ed.ac.uk

---

**Constructor Summary**

`CommandLineMenu(java.lang.String name,
java.lang.String programDescription,
java.util.ArrayList<CommandOption> options,
java.lang.String exampleCommand,
java.lang.String authors)`


| **Method Summary** | |
| --- | --- |
| `java.lang.String` | `toString()`             "-n  The file containing the network e.g. |

**Methods inherited from class java.lang.Object**

`equals, getClass, hashCode, notify, notifyAll, wait, wait, wait`

**Constructor Detail**

### CommandLineMenu

```
public CommandLineMenu(java.lang.String name,
                       java.lang.String programDescription,
                       java.util.ArrayList<CommandOption> options,
                       java.lang.String exampleCommand,
                       java.lang.String authors)
```


**Method Detail**

### toString

```
public java.lang.String toString()
```

:   "-n  The file containing the network e.g. in sif format (any Cytoscape format is valid http://www.cytoscape.org/)"
    + "\n" +
    "-o  File containing the node names to act as origin for the path search (one per line)"
    + "\n" +
    "-t  File containing the node names to act as targets for the path search (one per line)"
    + "\n" +
    "-r  The path and name of the report file to be created"
    + "\n" +
    "-s  e.g. \\s for space \\t for tab or , (comma). Default is tab separated."
    + "\n" +
    "-m method (algorithm) Either 0 = single shortest path (directed and undirected) or 1 = PESCA multi shortest path (all undirected shortest paths). Default is 0."
    + "\n" +
    "-a  (optional) The directory containing the .NA (node attributes) .EA (edge attributes) Cytoscape files."
    + "\n" + "-h > gets this help."+ "\n" +

    :   **Overrides:**: `toString` in class `java.lang.Object`


---


|  |  |  |
| --- | --- | --- |
| **Overview** \| **Package** \| **Class** \| **Use** \| **Tree** \| **Deprecated** \| **Index** \| **Help** | |  |
| PREV CLASS   **NEXT CLASS** | **FRAMES**    **NO FRAMES**     **All Classes** |  |
| SUMMARY: NESTED \| FIELD \| CONSTR \| METHOD | DETAIL: FIELD \| CONSTR \| METHOD |  |


---
